# Immunological and Technical Considerations in Application of Alginate-Based Microencapsulation Systems

**DOI:** 10.3389/fbioe.2014.00026

**Published:** 2014-08-06

**Authors:** Genaro Alberto Paredes Juárez, Milica Spasojevic, Marijke M. Faas, Paul de Vos

**Affiliations:** ^1^Section of Immunoendocrinology, Department of Pathology and Medical Biology, University Medical Center Groningen, University of Groningen, Groningen, Netherlands; ^2^Department of Polymer Chemistry, Zernike Institute for Advanced Materials, University of Groningen, Groningen, Netherlands

**Keywords:** alginate, purification, microencapsulation, PAMPs, DAMPs, innate immune activation, non-human models

## Abstract

Islets encapsulated in immunoprotective microcapsules are being proposed as an alternative for insulin therapy for treatment of type 1 diabetes. Many materials for producing microcapsules have been proposed but only alginate does currently qualify as ready for clinical application. However, many different alginate-based capsule systems do exist. A pitfall in the field is that these systems are applied without a targeted strategy with varying degrees of success as a consequence. In the current review, the different properties of alginate-based systems are reviewed in view of future application in humans. The use of allogeneic and xenogeneic islet sources are discussed with acknowledging the different degrees of immune protection the encapsulation system should supply. Also issues such as oxygen supply and the role of danger associated molecular patterns (DAMPS) in immune activation are being reviewed. A common property of the encapsulation systems is that alginates for medical application should have an extreme high degree of purity and lack pathogen-associated molecular patterns (PAMPs) to avoid activation of the recipient’s immune system. Up to now, non-inflammatory alginates are only produced on a lab-scale and are not yet commercially available. This is a major pitfall on the route to human application. Also the lack of predictive pre-clinical models is a burden. The principle differences between relevant innate and adaptive immune responses in humans and other species are reviewed. Especially, the extreme differences between the immune system of non-human primates and humans are cumbersome as non-human primates may not be predictive of the immune responses in humans, as opposed to the popular belief of regulatory agencies. Current insight is that although the technology is versatile major research efforts are required for identifying the mechanical, immunological, and physico-chemical requirements that alginate-based capsules should meet for successful human application.

## Introduction

Patients suffering from diabetes would benefit from an endocrine insulin source that regulates glucose metabolism on a minute-to-minute level. A minute-to-minute regulation avoids frequent episodes of hyperglycemia and hypoglycemia as with exogenous insulin therapy, and therefore, improves the quality of life (Vaithilingam and Tuch, 2011). Therapeutically, there are two options for transplanting an endocrine insulin source, i.e., transplantation of the whole pancreas or transplantation of only pancreatic islets. Pancreas transplantation is currently being performed in almost all major surgical centers but requires major surgery, life-long immunosuppression, and is associated with morbidity (Vaithilingam and Tuch, 2011). For these reasons, pancreas transplantation is only applied in diabetics that suffer from end-stage renal failure and receive a combined kidney and pancreas as a life-saving intervention.

Transplantation of pancreatic islets is not associated with major surgery as it involves a small amount of tissue and is, therefore, considered to be a better option for diabetic patients (Mittal et al., 2014). Another advantage of transplantation of pancreatic islets is their size. Islets are small organs of 50–350 μm that can be manipulated in order to prevent rejection. Currently, however, islet-transplantation requires chronic application of immunosuppressive therapy that restricts the use of this technique (Ryan et al., 2005; Figliuzzi et al., 2014). Up to now, only patients with unstable metabolic control, repeated severe episodes of hypoglycemia and hypoglycemic unawareness, or those with rapidly progressive diabetes-associated complications are eligible for islet-transplantation in most centers (Vantyghem et al., 2014). This will change with new technical approaches that minimize or completely prevent rejection of islets.

Application of immunosuppressive medication can be avoided when islets are enveloped in immunoprotective membranes. These membranes protect pancreatic islets from the effector side of the host immune system and thus prevent rejection. This encapsulation in immunoprotective membranes can be done in two geometries, i.e., macro- and microcapsules. In macrocapsules, the cells are packed in relatively large diffusion chambers. The walls of these chambers are semipermeable. Macrocapsules can be applied as intra- or extravascular devices. In intravascular approaches, the cells are seeded outside of artificial capillaries and connected to the blood stream. The advantage of intravascular devices is close contact of cells with the blood stream implying the fast exchange of glucose and insulin. A major disadvantage, however, is that thrombosis may occur. This makes the use of life-long anti-coagulation therapy a requirement (Uludag et al., 2000; de Vos et al., 2006a; Krishnamurthy and Gimi, 2011; Nafea et al., 2011; Vaithilingam and Tuch, 2011). For diabetic patients, this risk of thrombosis makes intravascular devices an unacceptable alternative for insulin therapy. For this reason, most groups currently focus on extravascular devices, in which pancreatic islets are enveloped within semipermeable diffusion chambers and implanted under the skin or in the peritoneal cavity without direct vascular access. The technology is associated with minor surgery and allows replacement of the graft when the islets have to be substituted.

**Figure 1:**
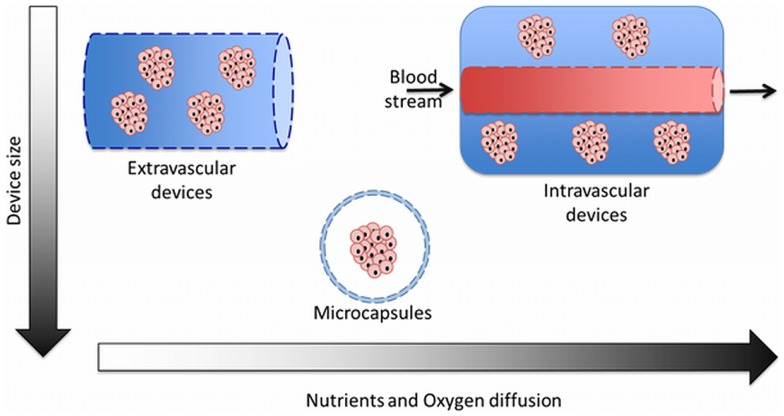
**The three different categories of encapsulation devices used for immunoisolation of islets**. Comparison between size and diffusion rate of nutrients and oxygen through the semipermeable membranes.

Microcapsules are very different in concept from macrocapsules. Within this technology, islets are individually packed in their own, individual capsule. An advantage over macrocapsules is that the microcapsules have an optimal surface to volume ratio, which implies a faster exchange of glucose, insulin, and nutrients (Uludag et al., 2000; Krishnamurthy and Gimi, 2011; Nafea et al., 2011; Vaithilingam and Tuch, 2011). For this reason, microencapsulation is preferred over macrocapsules as nutrient supply is more readily available in microcapsules than in macrocapsules. Especially, oxygen diffusion is an issue. Islets require relative high amounts of oxygen for both function and survival. Oxygen availability to the islets depends more on the O_2_ partial pressure (pO_2_) in the transplantation site rather than on O_2_ concentrations such as with other nutrient (Johnson et al., 2011; Colton, 2014) (Figure 1).

Microcapsules, however, can be obtained in many different forms, sizes, composition, and with different permeability (de Vos et al., 2014). Some use cation-crosslinked concepts while others use multiple layer systems (Lebedeva et al., 2004; de Vos et al., 2009; Dufrane and Gianello, 2012). The capsules are being used for allogeneic sources as well as for xenogeneic sources without taking into account that for xenogeneic sources other requirements have to be met than those for allogeneic sources. For protection of allogeneic tissue, the prevention of cell–cell contact between donor-islets and immune-cells is considered to be sufficient to prevent rejection (Duvivier-Kali et al., 2001). Simple systems such as cation-alginate capsules (Mazzitelli et al., 2008) can, therefore, be effective in protection for allogeneic responses. However, when the donor-recipient histocompatibility becomes more discordant such as with xenografts other processes come into play (Anderson and Kirk, 2013). When highly immunoreactive epitopes on xenogenic islets diffuse out of capsules, such as galactosyl residues, they may react with naturally occurring (anti-Gal) and non-Gal IgM antibodies. The previous may activate the classical complement pathway and may lead to neutrophil infiltration (Dufrane and Gianello, 2012) and release of deleterious cytokines that can pass the capsule membrane. IgM is not able to pass most of the capsule’s membrane but the IgM-mediated humoral reaction against xenogeneic epitopes can also induce the typical delayed-type hypersensitivity response associated with xenografts (Dufrane and Gianello, 2012), which leads to significant production of even smaller cytokines that can freely pass the membrane. These immunological processes in association with the type of microcapsule system will be discussed in the present review in view of future application for protection of grafts for small cytokines produced in several immunological responses that occur after implantation of microencapsulated pancreatic islets.

## Polymers for Microencapsulation

**Figure 2:**
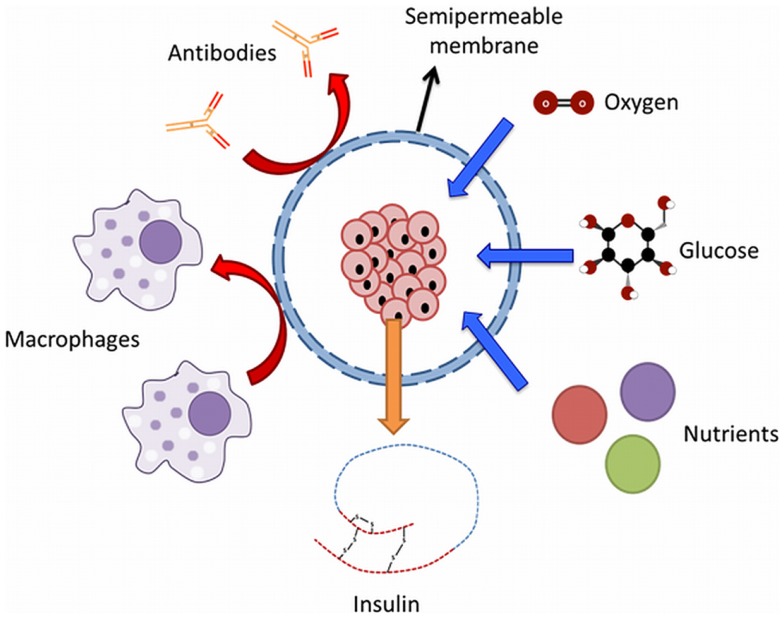
**Principle of immunoisolation by a microcapsule**. The semipermeable membrane allows the diffusion of molecules such as nutrients, glucose, oxygen, and insulin while protecting the graft from the effector molecules of the immune system of the host.

Polymers applied for microcapsules should meet a number of requirements, they should not negatively impact the viability of encapsulated cells, be flexible, soft, mechanically stable, allow diffusion of molecules of interest, and highly biocompatible to reduce host immune responses (Figure 2). The polymers that have been applied are derived from synthetic or natural sources. The most commonly used sources have recently been reviewed and are poly(ethylene glycol), polyvinyl alcohol, polyurethane, polyethersulfone, polypropylene, sodium polystyrene sulfate, polyacrylate, agarose, chitosan, cellulose, collagen, xanthan, and alginate (de Vos et al., 2014). The current consensus is that only alginate has been studied in sufficient detail to qualify as safe for human application (de Vos et al., 2014). Other polymers might become available in the future but have not been sufficiently studied in terms of compatibility with functional survival with the enveloped tissue and the host. Also diffusion characteristics of essential immunological molecules and nutrients have been poorly studied with other polymers. Therefore, when describing the immunological issues in the next sections and the different approaches of encapsulation, we will mainly concentrate on alginate as the core biomaterial. We would like to emphasize, however, that these are all generic issues that also other systems have to take into account.

## Different Alginates Results in Different Capsule Properties

**Figure 3:**
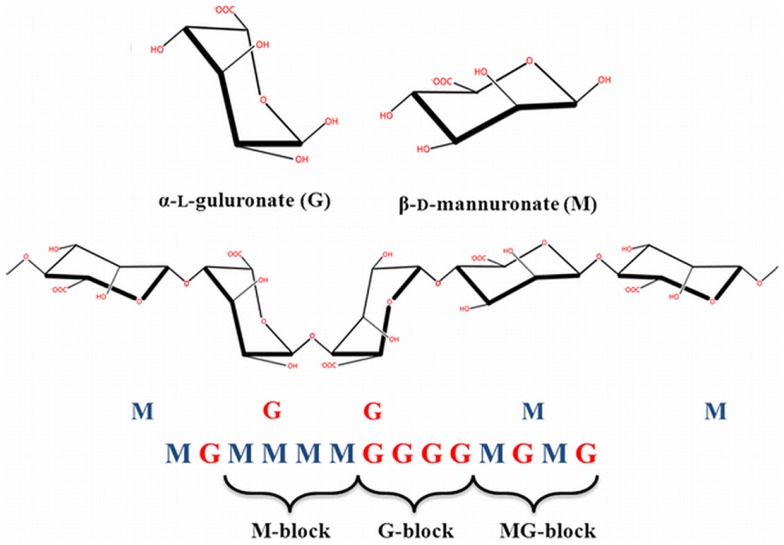
**Chemical structure of alginate**. Linear block polymers of β-d-mannuronate (M) and -l-guluronate (G) with a variation in composition and sequential arrangements.

**Figure 4:**
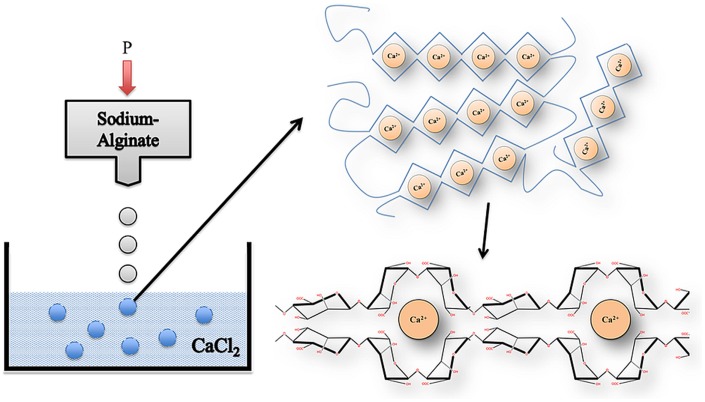
**Gelation process of alginate**. Alginate dropped from an air droplet generator into a CaCl_2_ solution to form a non-homogenous microcapsule in an “egg” formation. Gels are formed by cross-linking of alginate-polymers with calcium ions between G-G and M-G-blocks.

**Figure 5:**
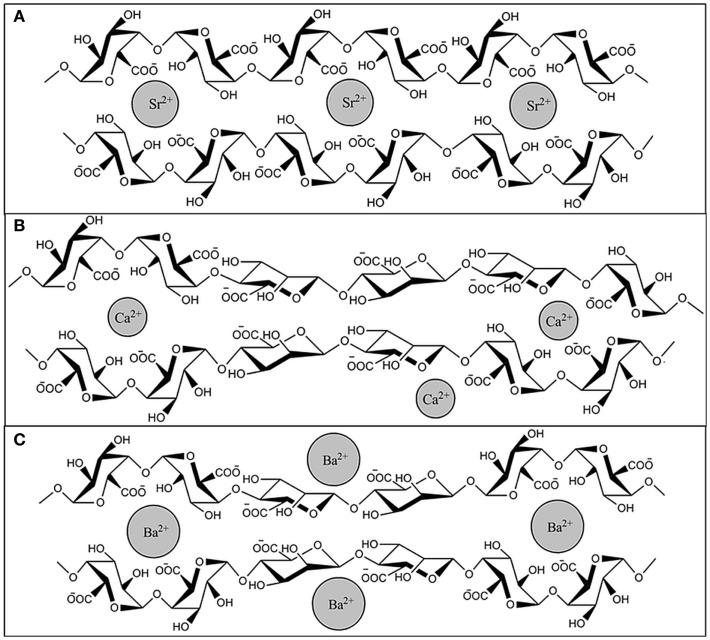
**Binding of Sr^2+^, Ca^2+^, and Ba^2+^ to alginate**. Cross linking between ions and G-G and M-M or G-M blocks of alginate occurs in specific conformations, **(A)** Sr^2+^ binds to G-G blocks solely, **(B)** Ca^2+^ binds to G-G and M-G blocks, and **(C)** Ba^2+^ binds to G-G and M-M blocks.

In 1881, Stanford was the first to describe alginate (Draget, 2000). Alginate is an unbranched binary copolymer of 1–4 linked β-d-mannuronic acid (M) and α-l-guluronic acid (G) (Figure 3) that can be isolated from algae like *Laminaria hyperborea*, *Macrocystis pyrifera*, *Laminaria digitata*, *Ascophyllum nodosum*, *Laminaria japonica*, *Eclonia maxima*, *Lessonia nigrescens*, *Durvillea antarctica*, and *Sargassum* spp. It can also be found as a polysaccharide in bacteria such as *Azotobacter vinelandii* and *Pseudomonades* (Wee and Gombotz, 1998; Draget, 2000). In principle, alginate is built of G-G blocks, G-M blocks, and M-M blocks. These blocks can be found in different ratios and in different molecular weights in alginate preparations, which gives them different physical and chemical characteristics (Ostgaard et al., 1993; de Vos et al., 2014). There are numerous types of alginates as a consequence. In the encapsulation-field, alginates are classified as high-G alginate, intermediate-G alginate, and low-G alginate. For forming microcapsules, alginates are usually collected in a solution containing high concentrations of cations. This leads to gel-formation. During this process, the uronic acid blocks in alginate bind to cations, like in an egg box model (Figure 4). The constitutive uronic molecules in alginate create zones in the gel that are interconnected. This provides rigidity. However, the rigidity and elasticity of the gels is not only determined by the type of alginate but also by the type of cation applied. This is influenced by the chemical properties of the cation applied such as atomic number, ionic radius, ionic strength, association constant, and chemical affinity toward alginate. The binding of ions is highly selective and the affinity strongly depends on the alginate composition and sequence. More specifically, Ba^2+^ binds to G-G and M-M blocks, Ca^2+^ binds to G-G and M-G blocks, and Sr^2+^ binds to G-G blocks solely (Figure 5). The amount of blocks determines the cross-linking degree, and therefore, also the rigidity and strength of the capsule. Also the permeability and, thus, the immunoprotective properties are determined by the type and concentration of the alginates in combination with the type of cation. The reason to mention and discuss these items explicitly is that many researchers in the field are not aware of these essential factors for capsule performance. This lack of awareness has led to the current extreme lab-to-lab variations in reported biocompatibility and immunoprotective properties of alginate-based capsules. This is a true obstacle for progress in the field as side-by-side comparison of data-sets is nearly impossible (de Vos et al., 2009, 2012; Mallett and Korbutt, 2009; Tam et al., 2011b).

## Polymers Might Contain Contaminations

To protect ourselves against harmful bacteria, viruses, and other microbes the human immunological defense system has developed two powerful weapons: the innate immune system and the adaptive immune system. The innate immune response is the first to act when uninvited hazardous elements invade our tissue. It is armed with powerful tools to attack invaders and to heal damaged tissue. This innate response is predominantly involved in foreign body responses. It reacts virtually in the same fashion against all type of foreigners and has almost no flexibility or memory. The adaptive immune response is more versatile but slow. It customizes its response to particular invaders and builds up an immunological memory after which life-long protection occurs against the foreign invader (Kawai and Akira, 2010; Mantovani et al., 2011; Netea et al., 2011; Kumar et al., 2013). The fast majority of encapsulation systems are designed to protect the tissue against the effects of the adaptive immune system (Jones et al., 2004). The relation and responses of the adaptive immune system have been reviewed in other papers (Franz et al., 2011; Mantovani et al., 2011; Oberbarnscheidt et al., 2011). The effect of the innate immune response on functional survival has not been reviewed intensively and has to our opinion not received the necessary attention.

**Figure 6:**
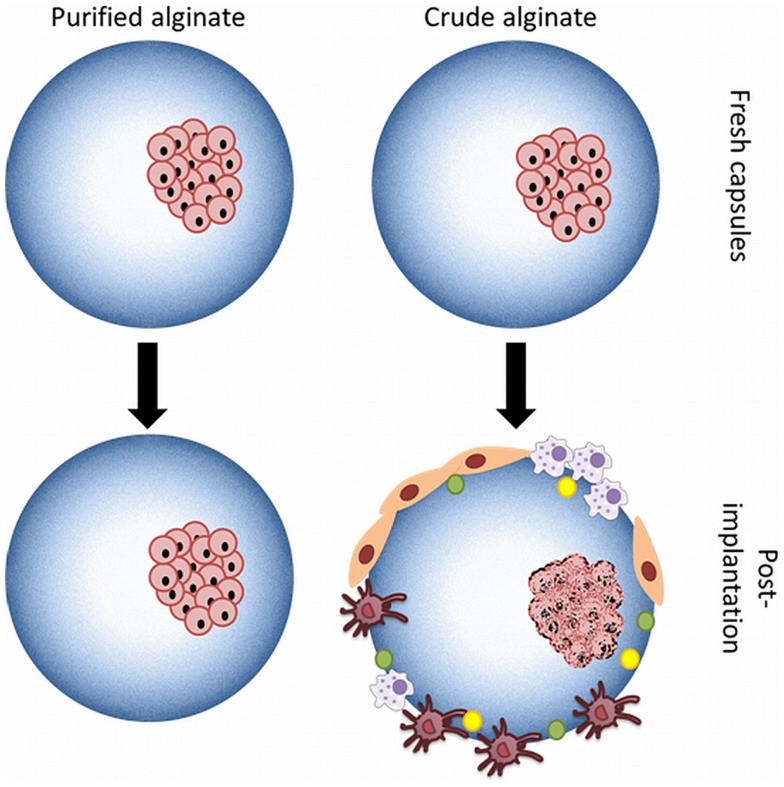
**Microcapsules produced with purified or crude alginate**. Use of crude alginate or alginates containing PAMPs promotes after implantation activation of the host immune system with recruitment of immune-cells as a consequence. Necrosis of the islet-cells is an inevitable consequence.

It is well recognized that when preparing alginate-based capsules with polymers the materials applied should lack any component that might provoke an innate immune system. The importance of applying true pure polymers has been demonstrated with alginate (Paredes-Juarez et al., 2013). It has been shown that crude alginates contain many components that provoke strong inflammatory responses leading to overgrowth of the capsule with immune-cells and fibroblasts (Figure 6). This overgrowth interferes with the exchange of nutrients and with necrosis of the islet-cells as a consequence. Removal of the immunogenic components from alginate has for these reasons been the focus of many research reports, but it is not a straightforward task and is still a struggle for many groups (de Vos et al., 1997; Kendall et al., 2004; Tam et al., 2006; Mallett and Korbutt, 2009; Ménard et al., 2010; Calafiore and Basta, 2014; Steele et al., 2014).

Alginates are isolated from natural sources and are contaminated with immunogenic substances during the industrial processes used for extraction. Impurities that have to be removed are molecules such as polyphenols, endotoxins, and proteins (de Vos et al., 2006b; Paredes-Juarez et al., 2014). By applying purification, a reduction of the host-response against the capsules can be accomplished but a persistent obstacle remains. Graft survival is never pertinent and varies considerable from several weeks to months (de Vos et al., 2006b).

It has become more and more recognized that immunogenic components in the polymers do not necessarily have to lead to overgrowth of the capsules. In almost all encapsulation systems, hydrogels are being applied, which are not readily compatible with cell adhesion. Immune-cells will only adhere when significant protein adsorption occurs or when anchoring points are available on capsule surfaces (de Vos et al., 2006b). In most cases, however, the inflammatory response will occur in the direct vicinity and not on the capsule’s surface (de Vos et al., 2002). It has been shown by our group (de Vos et al., 2002) that in the first days after implantation in rats significant amounts of immune-cells can be found in between the capsules but not on the surface of the capsules. This cell-infiltrate is composed of macrophages in different activation stadia, granulocytes, and basophiles (de Vos et al., 2002). These cells produce large quantities of cytokines, chemokines, and other deleterious bioactive molecules that can diffuse through most of the different types of capsules and kill islet-cells. Although the immune-cells have disappeared after 2 weeks, the reported loss of islets can be up to as much as 60% of the graft (de Vos et al., 2014). This all occurs without any adhesion of the cells to the capsule surface and can be missed if the responses against the graft are studied after period longer than 2 weeks (de Vos et al., 2002).

In search of factors contributing to this response, it was found that many alginates that are classified as pure and often recommended for human application still contain elements that provoke or contribute to the above described responses (Paredes-Juarez et al., 2013, 2014). Up to now these molecules were being addressed as endotoxins. The most commonly known endotoxin is LPS, but there are other molecules that also have to be qualified as endotoxins and which are responsible for many of the above-mentioned responses as will be outlined below. In a recent study, we have shown that commercially available “ultra-pure” alginates still contain lipoteichoic acid and sometimes even LPS (Paredes-Juarez et al., 2014).

## Pathogen-Associated Molecular Patterns in Polymer Preparations

A different word for endotoxins is pathogen-associated molecular patterns (PAMPs). In immunology-related research the term PAMPs is much more often used than endotoxin. The role of PAMPs in initiation of immune responses has been subject of intensive studies in the past decade and emerging new insight has been generated in how PAMPs induce activation of the innate immune system. The role of alginate-derived PAMPs in the responses against capsules in the immediate period after transplantation will be discussed in the next sections.

**Figure 7:**
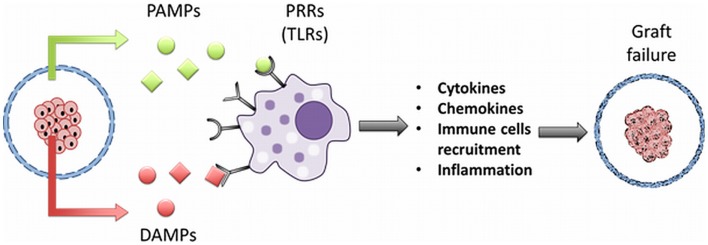
**PAMP and DAMP release from microencapsulated islets**. Impurities like pathogen-associated molecular patterns (PAMPs) in alginate can bind to pattern-recognition receptors (PRRs) such as Toll-like receptors (TLRs) on cells of immune system. This leads to release of cytokines, chemokines, chemotaxis, with graft failure as the ultimate consequence. Encapsulated islets under stress, release molecules required for cellular homeostasis but in the extracellular space act like danger or damage-associated molecular patterns (DAMPs). DAMPs can also bind to PRRs activating both the innate and adaptive immune system in the host.

Cells of the innate immune system that are involved in the reaction against capsules express innate receptors or sensors known as pattern-recognition receptors (PRRs) that can recognize evolutionary conserved molecules shared by pathogens (Kumar et al., 2013) but not present on mammalian cells (Oberbarnscheidt et al., 2011) (Figure 7). The PRRs are formed by receptor-families such as NOD-like receptors (NLRs), RIG-I-like receptors (RLRs), C-type lectin receptors (CLRs), DNA-sensing molecules, and Toll-like receptors (TLRs) (Hedayat et al., 2011; Kumar et al., 2013). The latter, the TLRs are well-known (Kawai and Akira, 2010, 2011; Kumar et al., 2013) and involved in responses against alginate-based capsules (Flo et al., 2002; Iwamoto et al., 2005; Paredes-Juarez et al., 2013). In humans, 10 different members of the TLRs family have been identified (Kumar et al., 2013) with specific cellular location and functions (Table 1). Mechanistically, most of TLRs interact with the coupling protein myeloid differentiation primary response gene 88 (MyD88), which leads to an internal signaling cascade that induces translocation of the transcription factor nuclear κB (NF-κB) from the cytoplasm to the nucleus (Pearl et al., 2011). Only two human TLR can signal in a MyD88 independent fashion, which are TLR3 that use the TIR domain-containing adapter inducing interferon-beta (TRIF), and TLR4 that can signal via TRIF or MyD88 (Pearl et al., 2011). In a recent study, we have shown that crude alginates contain flagellin, lipoteichoic acid, and peptidoglycans and that they cannot easily be removed (Paredes-Juarez et al., 2013, 2014). After purification alginates still contain lipoteichoic acid and some sources even LPS (Paredes-Juarez et al., 2014). All these molecules are recognized by TLRs as PAMPs (Table 1). When present in alginate, this will always lead to immune activation with cytokine release as a consequence (Paredes-Juarez et al., 2013, 2014). Most of these cytokines are small enough to pass the capsule membrane and will lead to cell-death (Juste et al., 2005; Nafea et al., 2011; Dufrane and Gianello, 2012). It should be mentioned that not only PAMPs but also small molecular poly-M residues should be removed. Poly-M has been shown to be a potent activator of TLR2 and 4 (Flo et al., 2002).

**Table 1:** **Human homodimeric toll-like receptors, PAMPs, and DAMPs that can be recognized by the different TLRs (Fritz and Girardin, 2005; Piccinini and Midwood, 2010; Basith et al., 2011; Rosin and Okusa, 2011; Liu and Ji, 2014)**.

TLR	Ligating pathogen-associated molecular patterns (PAMPs)	Ligating damage-associated molecular patterns (DAMPs)
TLR1	Triacy lipopeptides	β(-defensin-3
	Soluble factors	
TLR2	Zymosan	HMGB1
	Lipoteichoic acid	HSPs
	Lipoproteins	Monosodium uric acid
	Lipopeptides	Hyaluronan
	Peptidoglycan	Biglycan
	Bacterial porines	Versican
	Viric hemagglutinin	
	Glycolipids	
	Glycoinositol phospholipids	
TLR3	dsRNA	mRNA
TLR4	Lipopolysaccharides	HMGB1
	Taxol	HSPs
	Viral proteins	Monosodium uric acid
		Defensins
		Lactoferrin
		Hyaluronan
		Biglycan
		Fibrinogen
		Heparan sulfate
		Fibronectin extra domain A
		Envelope proteins
TLR5	Bacterial flagellin	Unknown
TLR6	Diacyl lipopeptides	Unknown
	Zymosan	
	Lipoteichoic acid	
	Phenol-soluble modulin	
TLR7	ssRNA	Cathelicidins
	Imidazoquinoline	
	Loxoribine	
TLR8	ssRNA	ssRNA
		Antiphospholipid antibodies
TLR9	Unmethylated CpG DNA	HMGB1
		DNA
		Cathelicidins
		IgG-chromatin complexes
TLR11	Uropathogenic bacteria	Unknown
	Profiling-like molecule	

## Danger Associated Molecular Patterns from Encapsulated Cells

As the consequence of the response described above, a significant portion of the islet-cells will die. However, more responses will occur in the immediate post-transplantation period that will lead to cell-death in the current encapsulation systems (Figure 7).

The implantation of grafts requires the application of surgery. Although, this is usually minor surgery, it still leads to a tissue repair response (de Vos et al., 2006b). A tissue repair response involves the release of essential bioactive wound healing promoting proteins such as histamine and fibronectin. The molecules prevent growth of bacteria and facilitate influx to the site of granulocytes, basophiles, and finally endothelial cells. How this response is modulated by the presence of the PAMPs in alginate is unknown but it is recognized that this response also contributes to loss of islet-cells (de Vos et al., 2006b). At the same time islet-cells start to produce islet-derived cytokines such as MCP-1 that attract even more immune-cells to the transplantation site. Dead cells in the capsule are the inevitable consequence.

Pattern-recognition receptors not only recognize PAMPs, but also intracellular components derived from dying cells in the capsules. These intracellular components function in a normal healthy organ as alarm signals that alert other cells of damage and promote restoration or healing caused by damage (Daly et al., 2012). These molecules are called danger or damage-associated molecular patterns (DAMPs) (Bianchi, 2007; Kono and Rock, 2008). DAMPs are well studied in aseptic or sterile inflammation (Hoque et al., 2012). This is an inflammation cascade not evoked by pathogens but induced by dying cell components that are recognized by PRRs, mainly via TLRs. Among these alarming molecules is high-mobility group box 1 protein (HMGB1) that in normal conditions plays a role in the winding of DNA and promotes assembly of proteins (Castiglioni et al., 2011; Daly et al., 2012). Another example of a DAMP that is involved in failures in clinical transplantation is heat shock protein 70 (HSP70). When it comes in the extracellular milieu HSP70 promotes recruitment of immune-cells (Garg et al., 2010; Piccinini and Midwood, 2010; Land, 2011). Other molecules that are studied and have been identified during sterile inflammation are uric acid and DNA (Rosin and Okusa, 2011). Currently, in transplantation-research, it becomes more and more recognized that DAMPs play a crucial role in the responses against grafts (Rosin and Okusa, 2011) and probably also plays a role in the responses against encapsulated islet grafts (de Vos et al., 2014).

## Design of Immunoprotective Microcapsules

As mentioned before, microcapsules have been designed to protect the enveloped cells against immune cell-donor cell interactions and high-molecular weight effector molecules of the adaptive immune system such as immunoglobulins and complement factors (Nafea et al., 2011). The capsules usually have a permeability that allow for the entry of molecules below 100 kDa and allow entry of large essential nutrient chaperons such as transferrin and lipid carriers (de Vos et al., 1997; Jones et al., 2004). Also insulin can diffuse readily out of the pores of the matrix. As a consequence most capsule systems do not protect against the bioactive molecules of the innate immune responses. The only effective strategy up to now has been to keep activation of the immune system as low as possible by applying alginates that lack any PAMP and by applying perfectly smooth surfaces that do not allow for cell adhesion and activation. It has been shown, however, that some microcapsule designs might partially protect encapsulated cells by interfering with diffusion kinetics of cytokines and chemokines (de Vos and Marchetti, 2002; de Vos et al., 2006b; Nafea et al., 2011). The final effect of the cytokines depends on their combined presence (de Vos and Marchetti, 2002). When studying the effects of the cytotoxic cocktail IL1-beta, TNF-alpha, and IFN-gamma, it has been shown that when the capsule network is dense, diffusion might be hampered, and partial protection might occur (de Vos et al., 2003). Diffusion of solutes through alginate does not only depend on the molecular weight of the molecule but also on the three-dimensional structure, the factor of gyration, and their charge density (Wee and Gombotz, 1998; de Vos et al., 2006b). TNF-alpha is a 50 kDa trimeric molecule that does not diffuse through the majority of microcapsule systems. Some capsule types containing an in-homogeneous core have even been shown to interfere with the diffusion of even smaller cytokines such as IL-1beta (17.5 kDa) (Kulseng et al., 1997). Applying or adapting the current microencapsulation systems might reduce cytotoxicity by innate immune effector molecules.

## Homogeneous and In-Homogeneous Core Capsules

**Figure 8:**
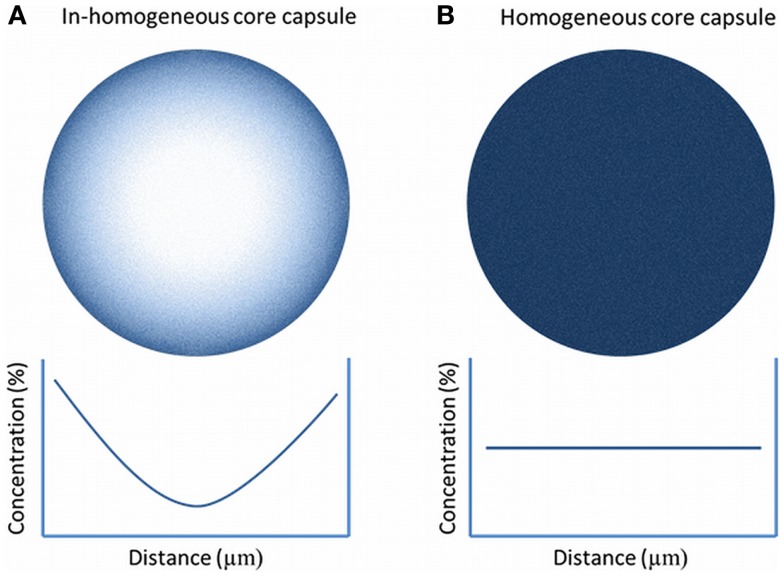
**Alginate gradient in in-homogeneous alginate-matrixes (A) and in homogeneous capsules (B)**. In in-homogeneous capsules, the concentration of alginates decrease gradually toward the center of the capsule while in the homogeneous capsule the alginate concentration is constant.

Microcapsules can be prepared with a homogeneous or an in-homogeneous core. Homogeneous beads have a similar if not identical concentration of alginate throughout the bead while non-homogeneous beads typically have a higher alginate concentration in the periphery and a lower concentration at the center of the bead (Figure 8) (Qi et al., 2008). In-homogeneous alginate gels can be produced by manipulating the diffusion of the alginate molecules in the bead. This can be done by substituting the ions in the gelling solution for iso-osmolites such as mannitol. The gelling solution should have a low divalent cation concentration. What subsequently will happen is that the alginate molecules will diffuse toward the surface of the bead in order to compete for the scarce divalent cations. The final consequence is a higher concentration of alginate-polymers on the surface and a lower concentration in the core. By lowering the cation concentration or increasing the polymer concentration a gradual increase of the alginate concentration at the rim of the capsule can be achieved (Mørch et al., 2006; Qi et al., 2008). This approach can be applied to manipulate the permeability properties of the capsules (Uludag et al., 2000) and might be an effective tool to modulate the diffusion of cytokines into the network.

**Figure 9:**
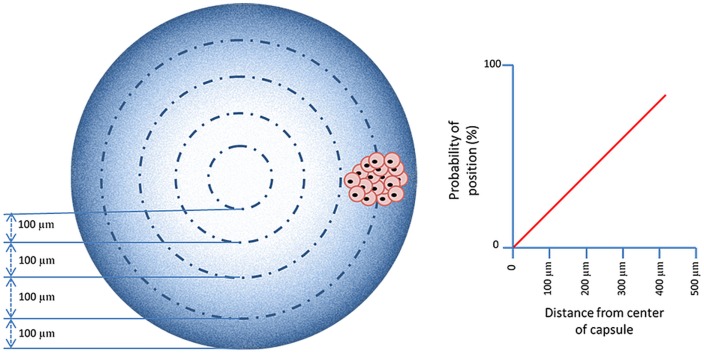
**The probability that islets are in the periphery of the capsules is higher than ending up in the center**. This is even more pronounced in in-homogenous alginate capsules as osmotic forces associated with movement of the alginate-polymers toward the surface induces a shift of the islets toward the periphery.

This elegant method has been applied (Qi et al., 2008; Safley et al., 2008) but has in addition to advantages also disadvantages. During formation of in-homogeneous gels, osmotic pressure is pushing the envelope tissue toward the rim of the capsules. This holds two risks. The first is that during this process significant shear forces are being put on the tissue. This may lead to fracture of the islets and loss of function. The second risk is the increased chance on protrusion of cells. The distribution of cells throughout a capsule is determined by chance. When a capsule with a radius of 500 μm is divided in zones of 100 μm, it can be calculated that the chance that a cell will end up in one of the outer rims is high (Figure 9). This is also confirmed by experimental studies (de Vos et al., 1994; Del Guerra et al., 2001). This implies that the cells will be pulled during the process of gel-formation toward the edge and can even protrude from the capsules. When using this method, it is advisable to apply assays to detect and/or remove capsules with protruding cells (de Vos et al., 1996).

Although a very elegant method, it still remains to be determined whether this system composed of alginate only can provide sufficient protection against the complex and inevitable activation of the innate and adaptive immune system. Especially, we have doubts whether the permeability and diffusion characteristics can be manipulated as such that protection is achieved against the harsh xenogeneic responses. To the best of our knowledge, there are no efficacy studies available of this system in non-immunosuppressed recipients.

### Barium beads

Homogeneous gels are still the most commonly applied form of gels in microcapsules. They have been applied in combination with different types of divalent cations (Safley et al., 2008). A popular one step system has been alginate-microcapsules gelled with barium. The microcapsules are characterized by a high mechanical stability (Tam et al., 2011a) and are easy to produce without many laborious complicated technological tools. The gel is formed after cross-linking of barium in solution with carboxyl groups of M-M and G-G chains (Zimmermann et al., 2005). Alginates with a high-G-content are preferred as these alginates bind barium to a higher extent than high-M alginates. It has been shown that barium-alginate encapsulated islets can restore normoglycemia in type 1 diabetic NOD mice for periods over 340 days (Duvivier-Kali et al., 2001). When these allografts were explanted, it was found that they lacked fibrotic overgrowth, and that islets in the capsules had a preserved secretory function (Duvivier-Kali et al., 2001). These results were not as successful when allografting was performed in rats, where the normoglycemia lasted not more than 12 weeks and parameters, such as C-peptide release were decreased (Omer et al., 2005).

When applying barium-beads for immunoprotection, it is mandatory to control a number of items. Barium can be toxic if present in high doses (Krishnamurthy and Gimi, 2011). It is associated with hypertension and renal toxicity (Mørch et al., 2012). It should be carefully controlled and documented that all non-bound barium is removed from the beads before implantation is considered. Also, the tissue in the capsule may suffer from barium toxicity. Gelation times should be as short as possible during cell encapsulation to avoid inhibition of potassium channels by barium in the encapsulated cells leading to cellular death (Zimmermann et al., 2005).

There is debate whether barium beads can protect against xenoresponses (Vaithilingam et al., 2011). The only way to reduce the permeability of barium beads is by increasing the alginate concentration and by using somewhat smaller alginate molecules (Mørch et al., 2006). This all can be done until a certain limit. When too high concentrations of alginate are applied, cells will be affected due to mechanotransduction (Huang et al., 2012). Mechanotransduction is a process in which cells sense that the stiffness of their surrounding is above a certain critical threshold (Orr et al., 2006). This leads to disturbance of cellular processes with cell-death as the final consequence. It is not sufficiently studied to which concentration the system can be build and stay safely under the mechanotransduction limit. It is, therefore, also not known what the permeability limit of barium-beads is.

### Polyaminoacid-alginate capsules

The most commonly applied microencapsulation system is still the alginate-polyaminoacid method. With this technique, an immunoprotective and stabilizing membrane composed of a polyaminoacid is built on the surface of an alginate bead. This has been done on both homogeneous and in-homogeneous alginate gels (Safley et al., 2008). An advantage of this system is that it is versatile and allows for easy adjustment of the permeability when for example the requirements are different for diverse tissues such as for allogeneic and xenogeneic tissue.

Among the most common applied polyaminoacid are poly-d-lysine (PDL), poly (ethylene glycol) (PEG), poly-l-ornithine (PLO), and poly-l-lysine (PLL), as well as diblock copolymers of PEG and PLL (Ponce et al., 2006). They have different properties that make them more or less appropriate for certain applications. Some groups prefer PLO coating as it is known to reduce the swelling and to increase the mechanical strength of alginate-microcapsules restricting diffusion of higher molecular weight molecules (Ponce et al., 2006). Others prefer PEG polymers to prevent potential protein adsorption on the surface of the capsules (Spasojevic et al., 2014). After three decades of research, there is still debate, however, on which polymer is most optimal to build an immunoprotective membrane. It should be noted that, just as with the choice of the core material of the capsules, there are many options, but only one polymer has been sufficiently studied to allow safe application as will be outlined below.

**Figure 10:**
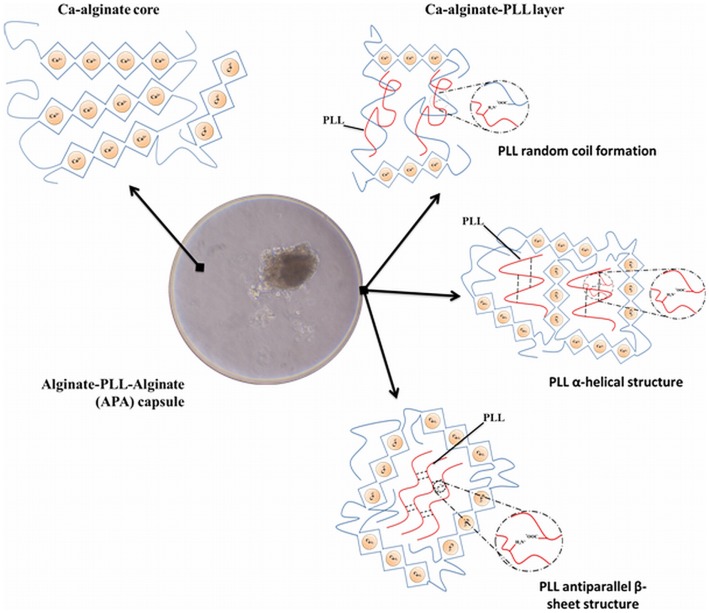
**Structure of alginate-poly-l-lysine capsules**. The system is composed of two layers and not of three layers as wrongly stated in many papers. The core of the capsule is composed of a matrix of alginate and calcium in which the islets are entrapped. The second layer is obtained after incubation of the calcium bead in sodium rich solutions to form a sodium-alginate complex at the surface. Sodium can be substituted by poly-l-lysine (PLL) to form a complex alginate-PLL- alginate (APA). The PLL-alginate layer can take three different structures by intramolecular hydrogen binding, (i) random coil formation between alginate and PLL, (ii) α-helicoidal structure between amide groups of PLL, and (iii) antiparallel β-sheet structure between amide groups of PLL.

A principle item that is missing in research on selection of adequate polyamino acids for reduction of permeability of microcapsules is mechanistic insight in how the molecules bind to alginate. This is extremely important as will be outlined for PLL. For many years, PLL was out of favor by researchers. The reason was several reports (Strand et al., 2001; Bünger et al., 2003) that described strong inflammatory responses against PLL membranes (Orive et al., 2006). When these reports are critically reviewed, it has to be concluded that the researchers were not taking into account the requirements for adequate PLL binding. Unbound PLL is cytotoxic and will have induced immune responses (Juste et al., 2005). To allow PLL binding, it is mandatory after gel-formation in the calcium-solution, to wash the beads with buffer with a low amount of calcium and high amounts of sodium. This step is required to extract calcium from the surface of the beads, which will subsequently be substituted by sodium. Sodium has a lower affinity for the alginate than PLL. The PLL, which is subsequently added, is binding to constitutive alginate molecules in a highly cooperative manner to form a strong, rigid membrane with the G-M molecules at the surface. This requires the binding of PLL with alginate in a superhelical core and beta-sheets (Figure 10). This process can be easily followed by Fourier-transform infrared spectroscopy (FT-IR) that has become an indispensable tool in understanding the interaction of polyamino acids on the capsule surface. The degree of cross-linking determines not only the mechanical stability and permeability of the membrane (van Hoogmoed et al., 2003) but also the biocompatibility. When PLL is not forced into the above-mentioned conformations, it will lead to strong inflammatory responses.

Unfortunately only the requirements for PLL binding to alginate-surfaces have been studied in sufficient detail to design a reproducible protocol. For other used molecules such as PLO (Calafiore, 2003), this has not been done as extensively. It is unknown to which alginate-polymer(s), the other polymers bind and in what chemical conformation they have to be to provide biocompatibility. It is impossible, therefore, to recommend a type of alginate for these polyamino acids. This was shown recently with the combination of alginate and PLL. In a comparison study of the *in vivo* behavior of a series of alginate-PLL capsules that differed only 10% in G-content, we observed strong inflammatory responses against one of the alginate-PLL membranes. This was due to detachment of the PLL with the alginate that did not contain sufficient amounts of G-M sequences required for persistent binding (de Vos et al., 2012).

To overcome biocompatibility issues with polyamino acids, some have chosen to apply an alternative strategy. They apply multilayer capsules in which the polyamino acid membrane is covered with an additional biocompatible alginate layer (de Haan et al., 2006). The polyamino acid is still used to reduce the permeability but its binding conformation has become less critical as the layer is not in direct contact with the host. Another strategy, which is developed by our own laboratory, is the application of diblock copolymers (Spasojevic et al., 2014). Diblock copolymers are molecules that are composed of two types of polymers that have different beneficial properties for the capsules. We tested and applied different types of PEG-b-PLL diblock copolymers. Binding of these copolymers was achieved through the ionic interactions between the positively charged PLL block and the negatively charged alginate. The other block, polyethylene glycol (PEG), should provide a biocompatible protecting layer on the surface of the capsules. Binding of the copolymer and the presence of the PEG blocks on the surface of the capsules was demonstrated in our previous study through the fluorescence labeling of methoxy end-group of PEG (Spasojevic et al., 2014). *In vivo* safety studies are in progress but the hope is that this concept may lead to more reproducible and less laborious procedures to reduce the permeability of alginate gels without interfering with biocompatibility.

In spite of the challenges that have to be met, the proof of principle of the alginate-polyamino acid system has been provided in both allogeneic and xenogeneic combinations and it has even entered the clinic (Calafiore et al., 2006b; Elliott et al., 2007). Calafiore et al. used microencapsulated pancreatic islets allografts in non-immunosuppressed patients with type 1 diabetes (Calafiore et al., 2006a,b). The grafts showed acceptable biocompatibility. However, insulin independence was not achieved as the authors were only allowed to use sub-optimal islet doses (Calafiore et al., 2006b). Also in rodents and dogs successes has been reported (Soon-Shiong et al., 1992; Tatarkiewicz et al., 2001). In non-human primates, the results are less favorable (He et al., 2013) but this might be due to differences in the immune system of the non-human primates as will be outlined in the next section.

## Are Primate Studies Predictable for Humans?

During recent years, there was a tendency to consider non-human primate transplantation studies as the ultimate proof for efficacy in humans (Anderson and Kirk, 2013; He et al., 2013). This reasoning is supported by arguments of the FDA (Merkel and Halperin, 2014) because of the assumption of similarities in immune responses between humans and non-human primates (Collins and Jones, 1978; Mezey et al., 1983; Vaithilingam and Tuch, 2011). Despite absence of scientific proof and meeting many ethical issues, the believers considered responses in non-human primates to be analogous to that in humans (Anderson and Kirk, 2013). To our opinion, this is a major misassumption. As will be outlined below, there are pertinent immunological differences between non-human primates and humans making the non-human primate, an inadequate model for human responses against encapsulated grafts. Immune responses may occur in non-human primates but will not occur in humans and specific innate responses not present in non-human primates may happen in humans.

Immunological differences are considered to be the reason for early mortality rates and life span rate differences between humans and non-human primates such as chimpanzees (Finch, 2010). Humans differ from chimpanzees by low mortality rates in both the juvenile and adult ages, and by the later onset of mortality rate acceleration, usually by chronic degenerative diseases (Finch, 2010). For wild chimpanzees, an early mortality rate of 20% per year in infancy is normal with a decrease to about 3.5% per year in pre-adult ages. Infections play a major role in the high chimpanzee mortality rates. The life expectancy at birth of a chimpanzee is about 13 years, whereas animals that reach adulthood have about 15 years of further life (Finch, 2010). This is very different in humans. Even in areas with limited access to medicine, life expectation after birth is much longer and the high early age mortality declines to a minimum at the approach of adulthood (Finch, 2010). There is consensus that this species difference in mortality rate and life span is due to differences in their immune system that were developed more than a million years ago (Finch, 2010; Anderson and Kirk, 2013).

Finch (2010) has done basic research in differences between humans and non-human primates innate and adaptive immune responses. Finch was interested in the variations in immune-genes that might explain the early differences in mortality rates between non-human primates and humans. To this end, Finch performed a stepwise comparison between chimpanzee and human-specific immunological genes and did some important discoveries. The most remarkable difference was in the composition of apolipoprotein E (ApoE) genes. This important gene is involved in metabolism of lipids and in regulating innate immune responses. There are three uniquely human ApoE alleles, of which ApoE e4 and ApoE e3 are the most prevalent (Schaffer et al., 2014). The ApoE e4 differs in several critical amino acids from the chimpanzee equivalent. It is the most relevant gene for the current discussion as it is responsible for boosting the human innate immune response, which is diminished in non-human primates relative to humans (Finch, 2010). ApoE e4 induces the acute innate response and initiates the production of important innate immune cytokines such as IL-6 and TNF-α (Vitek et al., 2009). Evolutionary, this gave humans the benefit to better fight harmful intruders that they ingested in food and encountered in their surroundings. This difference in innate immunity is considered to be a major factor in the difference in mortality rates in the juvenile and adult age classes between humans and chimpanzees (Barreiro et al., 2010). Also, this is pertinent when considering non-human primates as a relevant immunological model for humans since mimicking the human innate immune responses is a key in studies predicting the efficacy of encapsulated grafts in humans.

Besides differences in activity of the innate immune system, there are more immunological differences between humans and non-human primates that are relevant for the discussion on the adequacy of non-human primates as model for humans in our type of research. Humans lack for example specific genes or have specific polymorphisms making them incapable to perform some specific immunological responses. Major differences have been found in the major histocompatibility complex (MHC) (Finch, 2010). The MHC system is important for both innate and adaptive immune responses. An important species difference is the loss of polymorphisms in class IA and B genes in humans. This implies a class-specific loss of variation and therefore a selective elimination of responses against specific families of epitopes (de Groot et al., 2008). Also a pertinent difference in PRRs has been reported (Brinkworth et al., 2012). Human CD4^+^ T cells have low expression of Siglecs relative to chimpanzees (Nguyen et al., 2006). The Siglec lectin family of proteins (Ig superfamily) is involved in T-cell receptor (TCR) interactions and is implicated in the much milder chimpanzee symptoms of HIV-1 and hepatitis B or C (Bibollet-Ruche et al., 2008) infections due to more specific responses. This illustrates that non-human primates may respond differently and sometimes more rigorously against antigens than humans.

## Pre-Clinical Models

The foregoing obviously calls for suggestions for better animal models. The answer is simple. There is not a single step model that predicts efficacy of encapsulated grafts in humans. Technologies have emerged that has brought this research field into a next step were animal studies and *ex vivo* studies are combined to predict efficacy in humans. A first step is the need for animal models and *ex vivo* systems mimicking human innate responses and avoiding harsh innate immune responses is a key in the success of encapsulated islets in humans. Although abandoned by some (Anderson and Kirk, 2013; He et al., 2013) experimental animals such as mice and rats have a more developed innate immune response than non-human primates and are therefore more appropriate. Transgenic mice are available that resemble human innate responses as close as it can get. There are TRapoE4 mice available that have near identical human IL-6 and TNFα responses (Vitek et al., 2009). Also, technology platforms are under development containing human PRRs giving insight in how these sensors of the human immune system will react on encapsulated cell systems (Paredes-Juarez et al., 2013, 2014). Another advance and to our opinion mandatory research item is that some groups combine the foregoing studies with mimicking protein adsorption processes that occur in humans (de Haan et al., 2011; Rokstad et al., 2011). Protein adsorption to biomaterial surfaces is assumed to be the major cause of species differences (Vaithilingam and Tuch, 2011) in foreign body responses. It was shown by our group that the adsorption and changes at the capsule surface are highly dependent on the polymer applied and can be avoided (Spasojevic et al., 2014). This combined approach with a proof of principle study in any larger mammal with a well-developed innate immune system will be our preferred approach to come to a predictive outcome in humans.

## Future Consideration

As outlined in the preceding sections, there are many choices when considering alginate-based microcapsules for immunoprotection of pancreatic islets. Most of the systems mentioned above have shown some efficacy in allogeneic settings but in xenogeneic settings not many successful studies are available (Murua et al., 2009). This might not always be the consequence of immunological issues. Incompatibility in metabolism may also be a contributing factor. It has been shown, for example, that rat islets maintain their metabolism when transplanted in mice (Bobzien et al., 1983). This may lead to rapid exhaustion when the recipient has a higher metabolic demand than the donor. Another or combined explanation is that the prerequisites of the membrane are far more strict with xenografts than with allografts. Xenografts may provoke stronger responses by leaking immunoreactive epitopes, such as galactosyl residues, that may react with pre-existing, naturally occurring (anti-Gal) and non-Gal IgM antibodies with strong innate responses as a consequence. Currently, it is unknown whether the membranes can provide sufficient protection against the cytokines and deleterious bioactive molecules that are produced during xenoresponses (de Vos et al., 2014). It has been shown, however (de Vos and Marchetti, 2002), that human cytokines are not very effective in killing xenogeneic tissue due to structural differences in cytokine and receptor composition between humans and animals (Brinkworth et al., 2012). It is imperative for the future of encapsulation to determine, which of the above-mentioned factors contribute to the success or failure of immunoprotected xenografts and which sources have the capacity to maintain long-term graft function in humans.

Irrespective of the islet-source or system, it is mandatory to produce high-quality alginates that lack pro-inflammatory molecules such as PAMPs. PAMPs are introduced in alginates at different stages of production and can even be included during purification due to the application of chemicals or instruments that contain PAMPs (Paredes-Juarez et al., 2014). As outlined above all commercial alginates, even those that are marketed as “ultra-pure” and recommended for clinical application, do contain PAMPs and do initiate inflammatory responses (Paredes-Juarez et al., 2014). This is to our opinion, a major obstacle for clinical application of encapsulated islets. The ease of removal of PAMPs is in our hands alginate-type dependent. Removal of PAMPs from high-G alginates is more difficult than from intermediate-G sources. This is probably due to the fact that high-G alginate solutions have higher viscosities, which makes the washing steps during purification often less efficacious. Chemical extraction methods are most successful as well as dialysis against endotoxin free water. Combinations of these technologies as well as specific precipitation methods are probably required to remove PAMPs from alginate sources that are harsh to purify.

Stress signals released by microencapsulated islets themselves have not gained much attention in the success and failure of encapsulated islet grafts. As cell-death induced by necrosis is a major player in the failure of islet-grafts, it is plausible that DAMPs do contribute to the magnitude of the innate responses and failure of the grafts. A conceivable approach to overcome these types of issues, is the use of smaller islets (i.e., smaller than 150 μm) that can manage the relative low oxygen tensions without significant loss of cells by necrosis (Colton, 2014). Other strategies might be to design membranes that retain DAMPS or inclusion of pharmacological drugs such as necrostatin-1 that inhibits necrosis of β-cells (Tamura et al., 2011).

The above and most of the papers addressing species-specific efficacy of encapsulation systems discuss predominantly immunological issues. An overlooked but pertinent issue when comparing efficacy of encapsulation systems is the different requirements for mechanical stability, the capsules have to meet. It is unknown, for example, how rigid and elastic capsules should be in larger mammals. In rats, we know that capsules that can withstand a force of 8 g can survive up to 2 years in rats, i.e., the lifespan of rats. In pigs, however, the shear forces are much higher and more capsules were having imperfections on the membrane and contained immune-cells. This illustrates that, going from one species to another, adaptations have to be made in the mechanic-physic properties of the capsules and that caution should be taken to interpret the host-reactions as species-specific responses against the capsules. In our experience going from one organism to another requires significant adaptations of the system before any scientifically sound comparison in immune responses can be made (Scharp and Marchetti, 2014). Unfortunately, these types of adaptations are rarely done but are, to our opinion, mandatory now that encapsulation devices are coming closer to clinical application.

## Author Contributions

Genaro Alberto Paredes Juárez, Milica Spasojevic, Marijke M. Faas, and Paul de Vos contributed with conception and design of work and writing of the paper.

## Conflict of Interest Statement

The authors declare that the research was conducted in the absence of any commercial or financial relationships that could be construed as a potential conflict of interest.
